# T-Cell and Antibody Responses to Mycobacterial Antigens in Tuberculin Skin-Test-Positive *Bos indicus* and *Bos taurus* Cattle in Ethiopia

**DOI:** 10.1155/2012/457872

**Published:** 2012-05-20

**Authors:** Gobena Ameni, Paul Cockle, Konstantin Lyashchenko, Martin Vordermeier

**Affiliations:** ^1^Aklilu Lemma Institute of Pathobiology, Addis Ababa University, P.O. Box 1176, Addis Ababa, Ethiopia; ^2^Pfizer Vaccine Research, PGRD, La Jolla, San Diego, CA 92121, USA; ^3^Chembio Diagnostic Systems, Inc., Research and Development Department, 3661 Horseblock Road, Medford, NY 11763, USA; ^4^Animal Health and Veterinary Laboratories Agency, Weybridge, New Haw, Addlestone, Surrey KT15 3NB, UK

## Abstract

Higher IFN-**γ** responses to mycobacterial antigens were observed in *Bos taurus* (Holsteins) than in *Bos indicus* (Zebu) cattle which could due to differences in antigen recognition profiles between the two breeds. The present study was conducted to evaluate mycobacterial antigen recognition profiles of the two breeds. Twenty-three mycobacterial antigens were tested on 46 skin test positive (24 Zebu and 22 Holstein) using enzyme-linked immunospot assay (ELISPOT) and multiple antigen print immunoassay (MAPIA). Herds from which the study cattle obtained were tested for Fasciola antibody. The T cells from both breeds recognized most of the mycobacterial antigens at lower and comparable frequencies. However, antigens such as CFP-10, ESAT-6, Rv0287, Rv0288, MPB87, Acr-2, Rv3616c, and Rv3879c were recognized at higher frequencies in zebu while higher frequencies of T cell responses were observed to Hsp65 in both breeds. Furthermore, comparable antibody responses were observed in both breeds; MPB83 being the sero-dominant antigen in both breeds. The prevalence of Fasciola antibody was 81% and similar in both breeds. This piece of work could not lead to a definitive conclusion if there are differences in mycobacterial recognition profiles between the two breeds warranting for further similar studies using sound sample size from the two breeds.

## 1. Introduction

Historical reports indicated that *Bos taurus* (the group to which Holsteins belong) are more susceptible to bovine tuberculosis (TB) than *Bos indicus* (zebu) [1]. Experimental studies have also showed difference in susceptibility to bovine TB between *Bos taurus* and *Bos indicus *breeds [1]. It has also recently been found that there is higher prevalence of bovine TB in Holstein than in zebu kept under identical husbandry conditions in Ethiopia [2]. In addition, the severity of pathology in bovine TB was significantly higher in Holstein than in Zebu under a similar field cattle management in central Ethiopia [2]. Similarly, it was observed that IFN-*γ* responses to mycobacterial antigens were higher in Holstein than those in Zebu [3]. One of the possible reasons for the lower IFN-*γ* responses in Zebu could be a difference in antigen recognition profiles between Holstein and Zebu. Human and mouse studies have also shown that the immune response to particular mycobacterial antigens varies with the genetic background of the subjects involved [4, 5]. This hypothesis is being addressed in this study. To assess repertoire difference between Holstein and Zebu, peripheral blood mononuclear (PBMC) and sera from the two breeds were investigated for their specificities and intensities in respond to different mycobacterial antigens. 

## 2. Materials and Methods

### 2.1. Study Animals

For the assessment of T-cell responses 30-skin-test-positive cattle (14 Holstein and 16 Zebu) that were managed under the same field condition were used. These animals were recruited from herds in which the two breed types were kept under identical husbandry conditions by traditional farmers. They were screened by applying the single intradermal comparative skin test using avian PPD (aliquots of 0.1 mL of 2500 IU/mL; Veterinary Laboratories Agency, UK) and bovine PPD (aliquots of 0.1 mL of 2500 IU/mL; Veterinary Laboratories Agency, UK), and tested positive applying the standard interpretation of this test (responses to bovine PPD were more than 4 mm larger than those to avian PPD). Blood was collected 6 weeks after skin testing. For the evaluation of antibody responses, 16 cattle (8 Holstein and 8 Zebu) of similar age and having strong reactions to the TB StatPak lateral-flow assay (Chembio, NY, USA) were tested by multiantigen print immunoassay (MAPIA) as described earlier [6]. Although the cattle used for the evaluation of antibody were different from those cattle used the assessment of T-cell response, both groups of cattle were kept homogenously on pasture by the same traditional farmers in the same area. 

### 2.2. Mycobacterial Antigens

Avian and bovine PPDs were used for the skin test and *in vitro *immunological assays. Additionally, 23 mycobacterial antigens were used. Detailed information regarding these antigens is presented in Table 1.

### 2.3. Enzyme-Linked Immunospot (ELISPOT) Assay

The ELISPOT assay was performed following the procedure used by other researchers earlier [7]. Briefly, peripheral blood mononuclear cells (PBMCs) were isolated using ficoll-Histopaque-1077 (Sigma-Aldrich, Poole, UK) gradient centrifugation at 800 ×g for 45 min. The PBMCs were washed 2 times in HBSS (Gibco, Paisley, UK), supplemented with Heparin (Leo, Ballerup, Denmark) by centrifugation for 5 min at 500 ×g. The cells were resuspended at 2 × 10^6^ cell/mL in RPMI) 1640 medium supplemented with 10% foetal calf serum (FCS), penicillin/streptomycin, 2-mercaptoethanol, nonessential amino acids (all Sigma-Aldrich) (complete medium). Polyvinylidene difluoride (PVDF) microplates were coated at 4°C with 100 *μ*L/well (10 *μ*g/mL) anti-bovine IFN-*γ* capture monoclonal antibody (mAb) 5D10 (Bioscience, Wheatley, UK) diluted 1 : 600 in sterile coating buffer. The plates were then washed twice with RPMI-1640 and blocked with RPMI-1640/10% FCS for 2 h at 37°C. After removing the blocking solution, 100 *μ*L (2 × 10^6^ cell/mL) PBMC in complete medium was added into each well and stimulated with 100 *μ*L of each of the antigens at the concentrations indicated in Table 1. The plates were incubated at 37°C and 5% CO_2_ for 24 h and washed twice with distilled water and three times with phosphate buffer solution in Tween 20 (PBS-T, 0.05% Tween in PBS). Thereafter, 100 *μ*L rabbit anti-bovine IFN-*γ* diluted 1 : 100 in serum albumin (BSA, Sigma-Aldrich) was added into each well and incubated at room temperature for 1 h. The plates were then washed four times with PBS-T, which was followed by the addition of 100 *μ*L monoclonal anti-rabbit IgG alkaline phosphatase conjugated to streptavidin (Sigma clone R696 diluted 1 : 2000 in PBS-T/BSA) and incubated for 1 h at room temperature. Bromo-4-chloro-3-indolyl phosphate (BCIP) nitroblue tetrazolium (NBT) substrate was prepared by vortexing one buffered BCIP/NBT 6 substrate tablet (Sigma-Aldrich) in 10 mL distilled water 2-3 minutes. This was followed by the addition of 100 *μ*L substrate solution and incubation for 10 minutes in the dark. Thereafter, the substrate was flicked off, the plastic manifold was removed, and the plates were washed with water on the front and back. Then, the plates were air-dried and read using the automated AID ELISPOT Reader (AID, Strassberg, Germany).

### 2.4. Multiantigen Print Immunoassay (MAPIA)

MAPIA was performed as described previously [6] on sera of 16 cattle. A panel of 12 mycobacterial antigens were immobilized on nitrocellulose membranes (Schleicher & Schuell, Keene NH) at a protein concentration of 0.05 mg/mL using a semiautomated airbrush-printing device (Linomat IV, Camag Scientific Inc, Wilmington, DE). The membrane was cut perpendicular to the antigen pads into 4 mm wide strips. Strips were blocked for 1 h with 1% nonfat skimmed milk in PBS with 0.05% Tween 20 and then incubated for 1 h with serum samples diluted 1 : 50 in blocking solution. After washing, the strips were incubated for 1 h with horseradish peroxidase-conjugated protein G (Sigma) diluted 1 : 1000 for IgG detection or with peroxidase-labeled antibody to bovine IgM (Kikegaard and Perry Laboratories) diluted 1 : 500, followed by another wash step. Bovine antibodies bound to printed antigens were visualized with TMB membrane peroxidase substrate (Kirkegaard &Perry Laboratories, Gaithersburg, MD). Results were evaluated by visual observation for the formation of band against of the antigens used.

### 2.5. Antibody Detection for Fasciola

Fasciola seroprevalence was estimated in randomly selected 263 (67 skin test positives and 196 skin test negative) cattle. Sera samples were submitted to the Veterinary Laboratories Agency (VLA) for the examination for the presence of antibodies against Fasciolosis using enzyme-linked immunosorbent assay following the standard operating procedures of VLA.

## 3. Results

### 3.1. Mycobacterial Epitope Recognition Patterns of T Cells in Grazing Zebu and Holsteins

**Figure 1:**
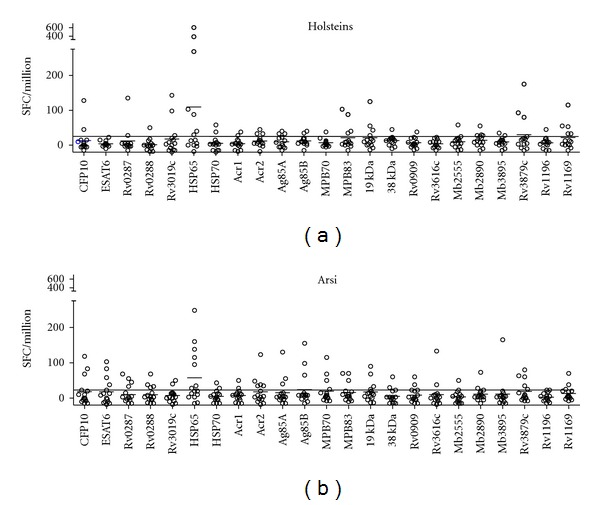
IFN-*γ* producing T cells. Enzyme immunospot assay (ELispot) was used for the assessment of IFN-*γ* producing T cell in response to novelmycobacterial antigens in 30 cattle. Twenty-three mycobacterial antigens were used to assess the response in each breed. Horizontal line = cutoff for positivity (25 SFC/million PBMC) used to calculate responder frequencies for figure.

ELISPOT analysis was performed for the assessment of the specificities and intensity of IFN-*γ* producing T cell in response to the defined mycobacterial antigens listed in Table 1.  Figure 1 shows T-cell responses of individual animals. Whilst hasp65 was recognized most frequently and most strongly, a number of additional antigens were also recognized at comparable frequencies by T cells from both breeds.

**Figure 2:**
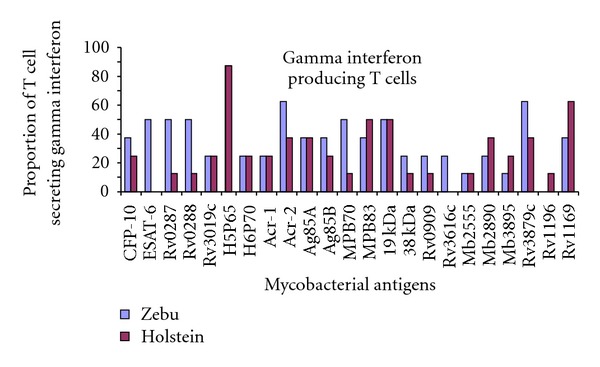
Proportion of IFN-*γ* producing T-lymphocytes in Holstein and zebu cattle. Enzyme immunospot assay (ELispot) was used for the assessment of IFN-*γ* producing T cell in response to novel mycobacterial antigens. Responder frequencies for Holstein and zebu cows were calculated using a cutoff for positivity >25 SFC/million). Although both breeds recognized the majority antigens studied at comparable frequencies, antigens such as CFP-10, ESAT-6, Rv0287, Rv0288, MPB87, Acr-2, Rv3616c, and Rv3879c were recognized at higher frequencies in Zebu than in Holstein.

When responses were stratified according to breed (Figure 2), although both breeds recognized the majority antigens at comparable frequencies, antigens such as CFP-10, ESAT-6, Rv0287, Rv0288, MPB87, Acr-2, Rv3616c, and Rv3879c were recognized at higher frequencies in zebu than in Holstein.

### 3.2. Mycobacterial Antigen Recognition Patterns of Antibodies in Grazing Zebu and Holstein

**Figure 3:**
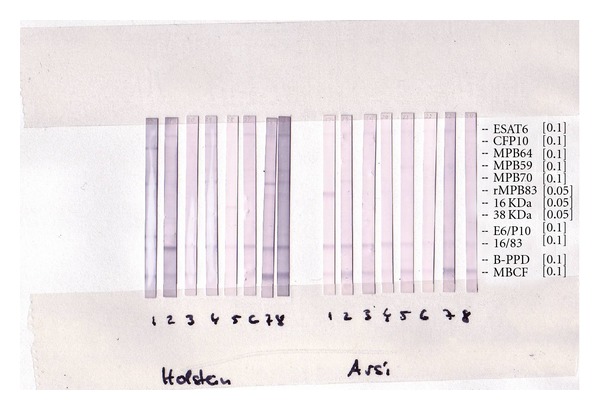
Antigen recognition pattern by antibody from Holsteins and zebus kept on pasture homogenously. Serological reactivity to *M. tuberculosis *complex antigens was determined by MAPIA for the two breeds. Antigens are indicated on the right margin, while the numbers on the bottom indicate animals (eight animals from each breed). *M. bovis *culture filtrate (MBCF) and 16 kDa alpha-crystallin/MPB83 fusion proteins (16/83) were serodominant antigens in both breeds. E6P10 refers to the ESAT6 : CFP10 fusion protein; PPDb refers to *M. bovis *PPD.

In order to investigate possible differences in antibody repertoire between Zebu and Holsteins cattle, MAPIA was performed. Holstein and Zebu exhibited similar antibody recognition profiles; with MPB83 being serodominant antigen in both breeds (Figure 3).

## 4. Discussion

In this study, the responses of T cell and antibody to mycobacterial antigens were compared in Holsteins and zebus kept under identical cattle management. In the ELISPOT assay, both Holsteins and zebus exhibited similar frequencies of T-cell responses to most of the mycobacterial antigens included in this study; a stronger T-cell response was observed to Hsp65 in both breeds. Previous studies [7] indicated that vaccination of calves with Hsp65 resulted in enhancement of lymphocyte proliferation following stimulation with Hsp65. The same workers have reported that PBMC from unvaccinated calves neither proliferate nor release IFN-*γ* following *in vitro *stimulation with Hsp65, whilst experimentally with *M. bovis*-infected cattle showed responses to this antigen [8, 9]. However, Hsp65 is an ubiquitously expressed antigen across the genus *Mycobacterium*, which could also result in cross-reactivity after exposure to environmental mycobacteria. However, higher responses to some of the specific antigens (ESAT-6, CFP-10, and MPB70) were observed in zebu than in Holstein. Nonetheless, as this experiment is preliminary, it needs further confirmation using sufficient number of cattle from both breeds.

In addition, a comparable recognition of mycobacterial antigens by antibodies from both Holstein and zebu breeds was also observed. *M. bovis *culture filtrate (MBCF) and 16 kDa alpha-crystallin/MPB83 fusion protein (16/83) were the serodominant antigens in both breeds. MBCF is a complex mixture of proteins and lipids, with a principal protein component of MPB70. The latter is a secreted antigen of *M. bovis *with high sequence homology with MPB83 [10, 11]. MPB70, ESAT-6, and CFP-10 were less frequently recognized by both breeds. A previous study [12] has also reported the sero-dominance of MBCF and 16/83 antigens in cattle infected with *M. bovis *experimentally. Waters et al. [12] further reported increment of the intensity of the antibody response to these antigens over time after inoculation of *M. bovis*. Similarly, in badger study, MBCF and MPB83 were the most frequently recognized antigens during infection with *M. bovis *[13].

However, in both breeds the intensities of the T-cell responses to mycobacterial antigens was generally low, which could be due to coinfection with helminthes that leads to a Th2 bias response thereby downregulating the T-cell response [14]. In our study, we found high seroprevalence of Fasciola in grazing cattle, which could result in generation of Th2 response resulting in inhibiting Th1 response [14]. Thus, Fasciola seropositivity was found in 81% (*n* = 213) in the population of cattle from which the study animals were selected. When these results were analyzed according to cattle breed, the prevalences of Fasciola antibody were similar (*χ* ^2^ = 4.26; *P* > 0.05) in Holstein (82.4%, *n* = 159) and in Zebu (78.8%, *n* = 104) cattle. Even within grazing cattle the higher IFN-*γ* response was reported in Holsteins compared to Zebu [3]. This could be attributed to the higher severity of pathology of bovine TB in Holsteins as compared to Zebu as observed in our earlier observation [2]. A positive correlation has been reported between the intensity of IFN-*γ* responses to mycobacterial antigens and severity of pathology of bovine TB under experimental conditions [15–17]. However, an ELISA readout was used in the previous study, and it is possible that the more responses were revealed in the more sensitive ELISPOT assay that we employed in the present study.

As this piece of work could not lead to a definitive conclusion if there is a difference in mycobacterial recognition profiles between Holstein and zebu cattle, further studies on the cytokine profiles and T-cell subset distribution in the two breeds are recommended.

## Figures and Tables

**Table 1:** List of mycobacterial antigens used the comparative study of immune response between *B. indicus* (Arsi breed) and *B. taurus *(Holstein) in Ethiopia.

Antigens	Protein	Peptides	Working concentration Final concentration at well (*μ*g/mL)	Supplier
(1)* ESAT-6-like: *				
CFP-10	X		5	Lionex^1^
ESAT-6	X		5	Lionex
Rv0288	X		5	Protix^2^
Rv0287	X		2	Dr Mustafa^3^
Rv3019c	X		5	Lionex
(2)* Heat-shock proteins: *				
HSP65	X		5	Lionex
HSP70	X		5	Lionex
Acr1	X		5	Lionex
Acr2	X		5	Protix
(3)* Secreted antigens/lipoproteins: *				
Ag85A	X		5	Lionex
Ag85B	X		5	Lionex
MPB83	X		5	Lionex
MPB70	X		5	Lionex
19 kDa antigen	X		5	Lionex
38 kDa antigen (Pst-1)	X		5	Lionex
(4)* Misc. *				
Rv0909		X	10	Pepscan^4^
Rv3616c		X	10	Pepscan
Mb2555	X		5	VSD^5^
Mb2890	X		5	Lionex
Mb3895		X	10	Pepscan
Rv3879c	X		5	VSD
Rv1196	X		2	Dr Mustafa
Rv1769	X		5	Lionex

^1^Lionex, Braunschweig, Germany; ^2^Protix Prague, Czech Republic; ^3^Dr A. Mustafa, Kuwait University, Kuwait; ^4^Pepscan, Lelystad, The Netherlands; ^5^Veterinary Science Division (VSD), Belfast, Northern Ireland.
